# Feasibility and effectiveness of telehealth in the management of cervicothoracic and lumbar pain during the first six months of the SARS-CoV-2 pandemic: A case series

**DOI:** 10.1016/j.inpm.2023.100260

**Published:** 2023-07-17

**Authors:** George Rappard, Jake Harb, Caitlin Yi, Robb Russell

**Affiliations:** a Los Angeles Minimally Invasive Spine Institute, 8929 Wilshire Blvd. Ste 210, Beverly Hills, California, 90211, USA; b Southern California University of Health Sciences, 16200 Amber Valley Dr, Whittier, California, 90604, USA

**Keywords:** SARS-CoV-2 pandemic, Telehealth, Neck pain, Low back pain, Conservative care, Interventional care

## Abstract

**Objectives:**

The primary study objective is to assess the effectiveness and utility of telehealth in managing spine pain. The secondary objective is to evaluate the feasibility of employing various treatments utilizing telehealth.

**Study design:**

Retrospective case series of patients with spinal pain managed primarily by telehealth during the first 6 months of the SARS-CoV-2 pandemic in the United States.

**Setting:**

A single center community based out-patient clinic and ambulatory surgical facility.

**Subjects:**

101 consecutive adult patients complaining of cervicothoracic or low back pain presenting to a specialized spine clinic.

**Methods:**

Telehealth was the preferred method of consultation for 101 consecutive patients presenting with cervicothoracic and/or low-back pain. After conservative care, patients with continued pain and disability were offered procedures. Disability Index (NDI and ODI) and pain Visual Analog Scores (VAS) were used to determine patient outcomes.

**Results:**

101 new out-patient consultations occurred. Telehealth initial consultation occurred in 98% of cases. There was a total of 504 follow up consultations. Follow up was via telehealth in 69%. Significant neurological abnormalities were detected by telehealth in 3% of patients. The lost to follow up rate was 10%. All 63 interventional procedures performed on 42 patients were completed as planned during telehealth visits. Likewise for all 9 surgical procedures. Outcomes were monitored via telehealth. Overall, for patients with cervicothoracic pain, minimal clinically important differences (MCID) for VAS or NDI were reached in 71%. Overall, the MCID for VAS or ODI for low back pain patients was reached in 70%.

**Conclusion:**

Telehealth in our series was easily deployable, highly feasible, allowed accurate monitoring of patient care and resulted in accurate triaging for interventions and surgery. Overall patient outcomes compare favorably with that reported for in-person spinal pain care. Telehealth was effective and easily utilizable.

## 1 Introduction

On January 30th, 2020 the World Health Organization declared the novel Corona virus outbreak originating in China a public health emergency. On March 13, 2020, the United States declared a national emergency in response to what had now become a pandemic brought on by the SARS-CoV-2 virus. The pandemic has brought on many changes to healthcare, among them an increased acceptance and utilization of telehealth [1]. Telehealth usage in orthopedic and spinal medicine had already begun to develop a body of evidence to justify its usage before the pandemic. Compared to face-to-face encounters telehealth was felt to possess high patient satisfaction rates [2], be cost effective [3,4], and have similar outcomes [5,6]. As a result, it comes as no surprise that telehealth was rapidly embraced by spine surgeons [[7], [8], [9], [10]]. Concerns regarding the telehealth adoption of the spinal physical examination were soon mitigated by the adaptation of new examination techniques [[11], [12], [13], [14], [15]] that were felt to be sufficiently accurate to justify their usage in determining care or planning procedures [[16], [17], [18], [19]]. As a result of medicine’s ability to adapt, telehealth had become a viable option to maintain patient care at the beginning of the pandemic.

The authors report on a large retrospective case series of spinal pain patients managed primarily by telehealth during the first 6 months of the SARS-CoV-2 national emergency in the United States. The primary study objective is to assess the effectiveness and utility of telehealth in the management of spine pain. The secondary objective is to evaluate the feasibility of employing various diagnostic and treatment modalities utilizing telehealth.

## 2 Methods

This study is a single center retrospective review of all patients presenting to a specialized spine treatment clinic with complaint of cervicothoracic (cervical and/or thoracic) or low back pain following the onset of the pandemic emergency in the United States. All patients presenting within the first six months of the pandemic are included in the study. Patients exited the study at discharge or after being lost to follow-up.

Patients presenting with cervicothoracic and low-back pain were managed with conservative care consisting of medications, activity restrictions, home exercise and, when possible, therapy. Patients with continued pain and disability were offered escalated care. Patients with suitable improvement after escalated care were discharged. Patients refusing escalating treatment recommendations after failing more conservative care were considered discharged.

Our clinic utilized SecureVideo as a telehealth platform and Secure Forms for demographic and questionnaire data collection. (Luxsci. 6 Liberty Square. Boston, MA. USA). SecureVideo and Secure Form are HIPAA-compliant, secure and encrypted solutions. Patients were required to sign a telehealth consent prior to their appointment. Patients required computer or smart phone access and an internet connection. A private venue was required for the patient to undergo telehealth evaluation.

Telehealth was the preferred method of consultation during the study duration. A standard history was performed using telehealth technology. A specialized observational examination was developed to evaluate telehealth patients presenting with cervicothoracic or low-back complaints (Table 1). An assessment and plan were conducted as usual. Patients with the inability to use telehealth technology were given the option of a live in-person consultation. Patients suspected of having neurological abnormalities were offered an urgent in-person consultation.

**Table 1:** Telehealth observational spinal examination.

General examination	Range of motion examination	Neurological examination	Special tests
Appearance	Neck extension	Shoulder height	Pronator drift
Orientation	Neck flexion	Shoulder shrug	Rhomberg's test
Facial symmetry	Neck rotation	Anti-gravity testing	Five-repetition sit-to-stand
Conjugate gaze	Neck lateral bending	Arm abduction	
Speech	Lumbar flexion	Arm flexion	
Affect	Lumbar extension	Arm extension	
Gait	Lumbar rotation	Hip flexion	
Posture	Lumbar lateral bending	Hip extension	
Balance		Leg extension	
Single leg		Hand	
feet side by side		1st and 2nd digit apposition	
Instep to toe		Supination	
Heel to toe		Pronation	
		Finger flexion	
		Finger extension	
		Lower extremity	
		Single leg standing	
		Heel walking	
		Toe walking	

Most patients underwent follow-up evaluation, performed preferentially using telehealth. All patients were seen for a mandatory live in-person follow-up prior to a planned intervention, a pre-surgical or a post-surgical visit. When live in-person visits were conducted in patients who previously had only been seen virtually, an appropriate comprehensive examination was carried out. This included palpation, range of motion testing, provocative maneuvers and a complete spinal neurological examination of the spinal segment in question.

On each visit questionnaires were scored. Cervicothoracic pain patients filled out the Neck Disability Index (NDI) questionnaire and completed visual analog scales (VAS). Low-back pain patients filled out the Oswestry Disability Index (ODI) questionnaire and completed VAS. NDI, ODI and VAS data were used to determine patient outcome at the conclusion of the study. Patients were considered to possess meaningful pain if their VAS scores were at least 5 out of 10 [20]. The minimal clinically important difference (MCID) utilized to determine treatment success based on VAS scores was considered to be a drop in score of 2 [[21], [22], [23]]. While scores of 0%–20% are considered mild disability on ODI [24] and scores of 5%–14% are considered mild disability on NDI [25], we chose to include patients with a threshold score of at least 10% to facilitate measurement of the MCID [21,[25], [26], [27], [28]]. Patients were considered an overall treatment successes if they reached MCID for VAS or NDI/ODI.

This study was exempt from Institutional Review Board (IRB) approval per institutional policy as only anonymized data was utilized in performing outcomes analysis. As such identifiable subjects are not involved in this study and the study was conducted in keeping with established ethical considerations in the study of human subjects [29,30].

## 3 Results

### 3.1 Patient characteristics, flow and lost-to-follow up

From March 2021 to September 2021, 101 consecutive patients presented for the evaluation of spinal pain. At presentation, and on follow up, study data was collected as described above [31]. Of these, 47 were male and 54 were female. Patient ages ranged from 18 to 80, with a mean age of 42. 89% (90/101) of patients were aged 60 or below and 48% (49/101) were between 20 and 40 years old. The mean time to presentation was 153 days from symptom onset. 56% (57/101) of patients presented within 12 weeks of symptom onset. 78% (79/101) of patients were categorized as having cervicothoracic pain, 86% (87/101) with pain related to the low back and 68% (69/101) with concurrent cervicothoracic and lumbar pain. The mean treatment duration was 79 days.

The patient population had symptoms predominantly post-traumatic in origin. 86% (87/101) sustained injuries as a driver or a passenger involved in a motor vehicle accident. 11% (11/101) were injured in falls. 2% (2/101) were pedestrians who were struck by automobiles and 1 was a bicyclist struck by an automobile. A single patient presented with spontaneous back pain after bending over.

**Fig. 1:**
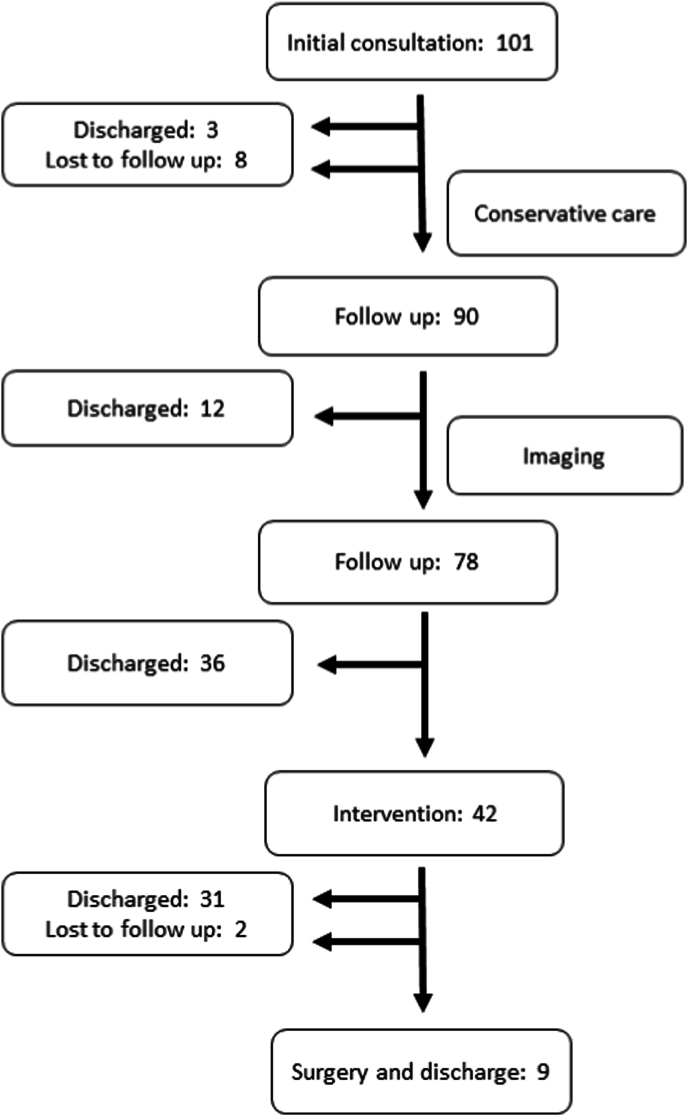
Patient treatment flow.

Patient flow during the study is shown in Fig. 1. Initial consultations were performed virtually in 98% (99/101) of cases. 93 patients underwent 504 follow-ups. Follow-up was via telehealth 69% (348/504) of the time. Telephonic follow-up occurred 5% (23/504) of the time. In-person follow-up (26% (133/504)) occurred prior to procedures, for wound checks, and in those with technology related difficulties. In-person follow-up also occurred in 3 patients noted to have significant neurological deficits on the initial tele-health consultation.

After the second follow-up visit the remaining patients underwent interventional procedures. All interventional and surgical procedures were planned during a telehealth follow-up. All procedures were completed as planned. The total lost to follow-up rate was 10% (10/101). These patients were regarded as treatment failures.

### 3.2 Treatment results: Cervicothoracic pain patients

**Fig. 2:**
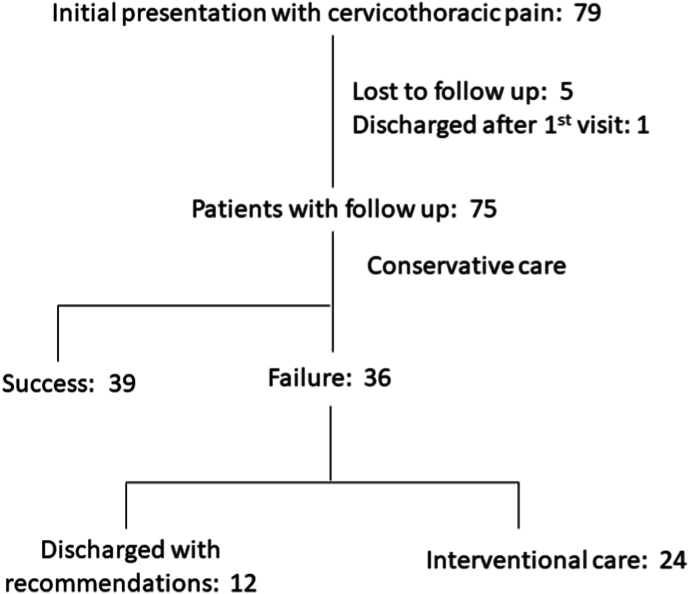
Cervicothoracic pain treatment flow.

**Fig. 3:**
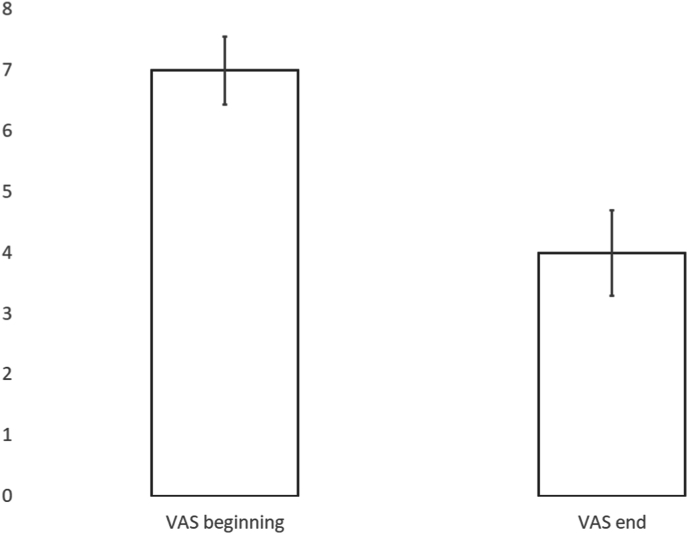
Mean Visual Analog Scores (VAS) in cervicothoracic pain patients.

**Fig. 4:**
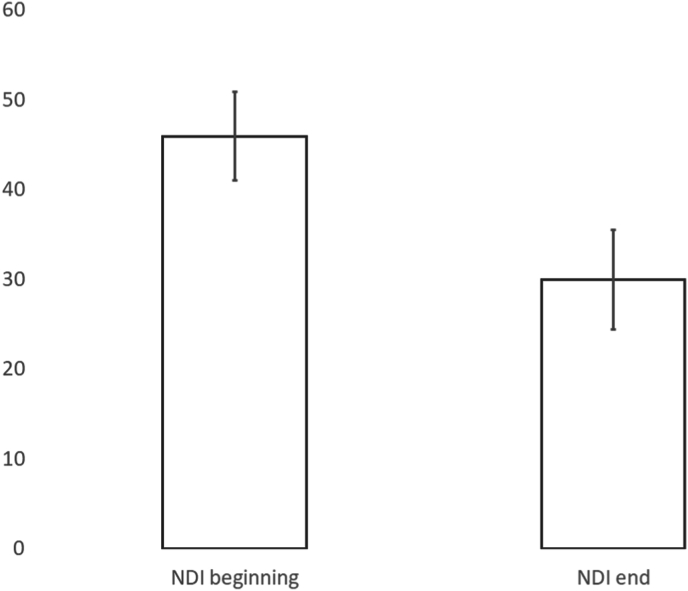
Mean Neck Disability Index (NDI) scores in cervicothoracic pain patients.

**Fig. 5:**
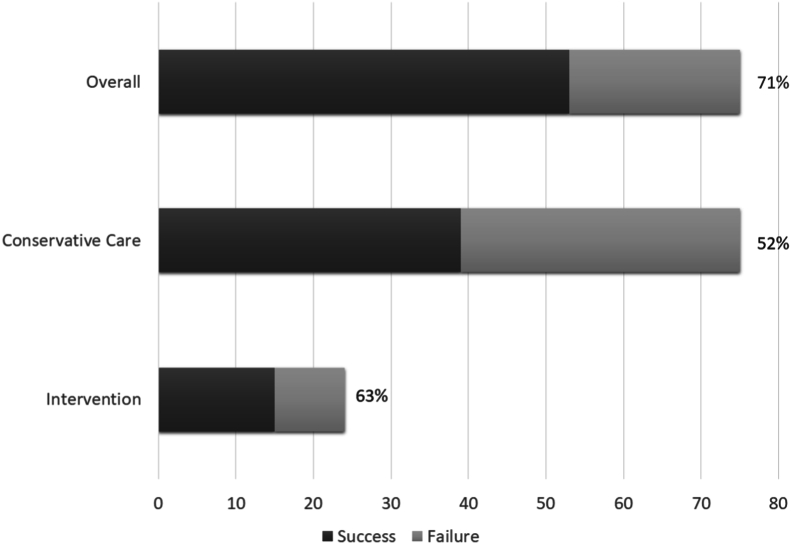
Cervicothoracic patients reaching MCID for VAS or NDI overall, after conservative care and after interventional procedures.

There were 75 patients presenting with cervicothoracic pain who underwent follow-up. All 75 underwent a period of conservative care. 51 patients were treated with conservative care only and 24 patients underwent interventional procedures (Fig. 2). The average presenting VAS was 7, 95% CI [7, 8], with 85% (64/75) of patients presenting with VAS ≥5. After treatment, the average VAS was 4, 95% CI [3, 5], (Fig. 3). The average presenting NDI was 46, 95% CI [41, 51], with 87% (65/75) of patients presenting with NDI ≥20. After treatment, the average NDI was 30, 95% CI [24, 36], (Fig. 4). MCID for VAS was reached in 57% ((43/75), 95% CI [46,69]), MCID for NDI was reached in 55% ((41/75), 95% CI [43, 66]), MCID for VAS and NDI was reached in 40% ((30/75), 95% CI [29, 51]) and MCID for VAS or NDI was reached in 71% ((53/75), 95% CI [60, 81]), (Fig. 5).

With conservative care, the MCID for VAS was reached in 39% ((29/75), 95% CI [28, 50]), the MCID for NDI was reached in 29% ((22/75), 95% CI [19, 40]), the MCID for VAS and NDI was reached in 19% ((14/75), 95% CI [10, 27]) and the MCID for VAS or NDI was reached in 52% ((39/75), 95% CI [41, 63]), (Fig. 5). 17% (13/75) of patients were discharged as conservative care failures; 10 with recommendations for interventional procedures and 3 with conservative care recommendations. The remaining patients underwent interventional procedures.

24 patients who did not improve with conservative care underwent interventional procedures. In these patients, the MCID for VAS was reached in 54% ((13/24), 95% CI [34, 74]), the MCID for NDI was reached in 54% ((13/24), 95% CI [34, 74]), the MCID for VAS and NDI was reached in 46% ((11/24), 95% CI [26, 66]) and the MCID for VAS or NDI was reached in 63% ((15/24), 95% CI [43, 82]), (Fig. 5). Of the 9 patients not reaching VAS or NDI success, 1 was lost to follow-up and presumed to have failed, 7 underwent diagnostic blocks without undergoing further recommended radiofrequency neurotomy and 1 was recommended to undergo surgery after failed cervical epidural injections.

### 3.3 Treatment results: Low back pain patients

**Fig. 6:**
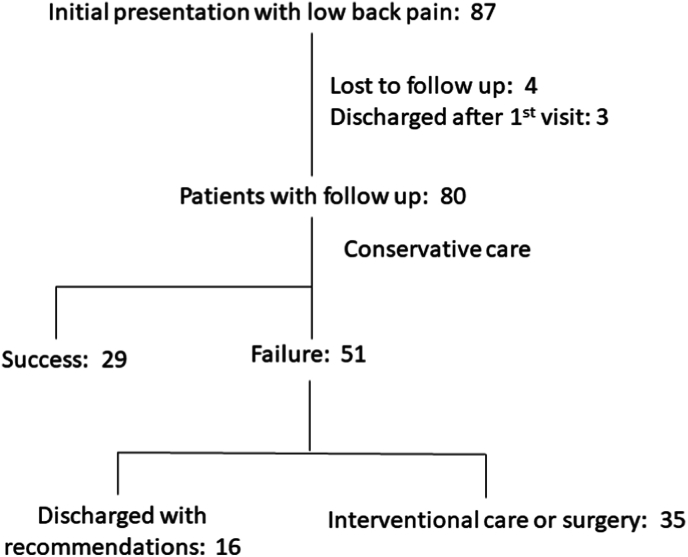
Low back pain treatment flow.

**Fig. 7:**
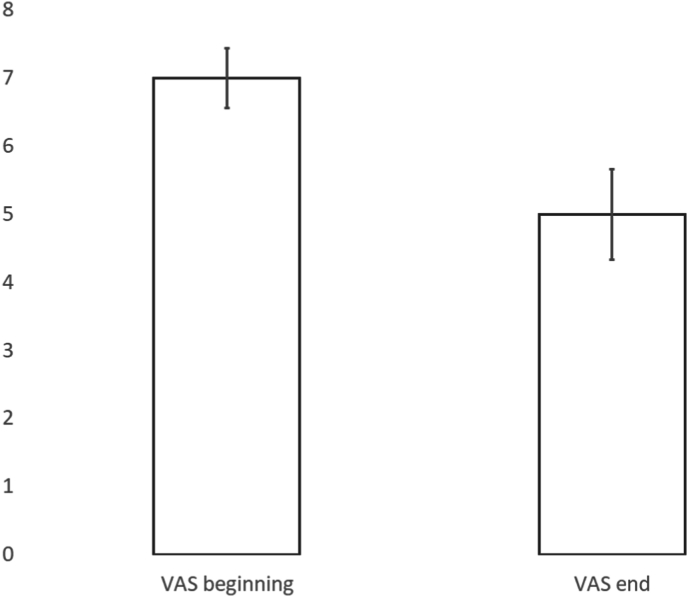
Mean Visual Analog Scores (VAS) in low back pain patients.

**Fig. 8:**
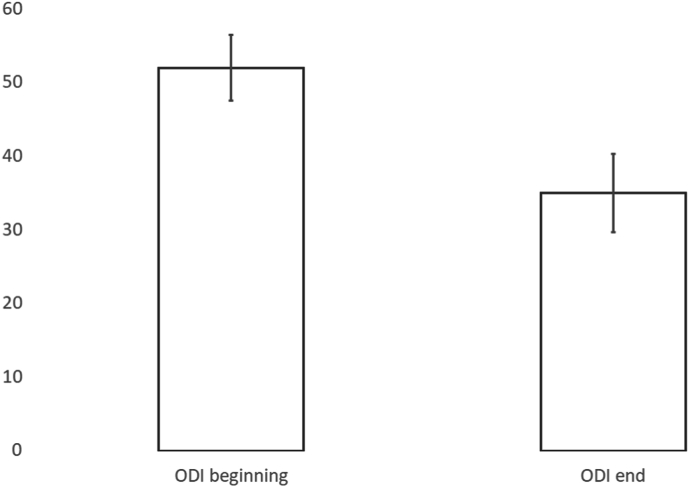
Mean Oswestry Disability Index (ODI) scores in low back pain patients.

**Fig. 9:**
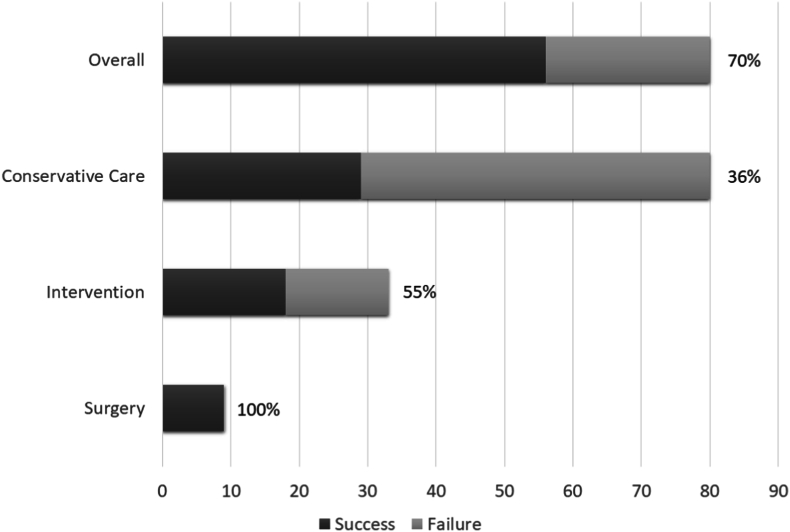
Low back pain patients reaching MCID for VAS or NDI overall, after conservative care, after interventional procedures and after surgery.

There were 80 patients with low back pain who underwent follow-up. 45 patients were treated with conservative care and 35 underwent interventional procedures. 9 interventional patients underwent lumbar surgery (Fig. 6). The average presenting VAS was 7, 95% CI [6, 7], with 91% (73/80) of patients presenting with VAS >5. After treatment, the average VAS was 5, 95% CI [3, 5] (Fig. 7). The average presenting ODI was 52, 95% CI [48, 56], with 95% (76/80) of patients presenting with ODI ≥20. After treatment the average ODI was 35, 95% CI [30, 40], (Fig. 8). MCID for VAS was reached in 58% ((46/80), 95% CI [47, 68]), MCID for ODI was reached in 60% ((48/80), 95% CI [49, 71]), MCID for VAS and ODI was reached in 48% ((38/80), 95% CI [37, 58]) and MCID for VAS or ODI was reached in 70% ((56/80), 95% CI [60, 80]), (Fig. 9).

With conservative care, the MCID for VAS was reached in 30% ((24/80), 95% CI [20, 40]), the MCID for ODI was reached in 31% ((25/80), 95% CI [21, 41]), the MCID for VAS and ODI was reached in 23% ((18/80), 95% CI [13, 32]) and the MCID for VAS or ODI was reached in 36% ((29/80), 95% CI [26, 47]), (Fig. 9). 20% (16/80) of patients were discharged as conservative care failures; 13 with recommendations for interventional procedures and 3 with conservative care recommendations.

35 patients who did not improve with conservative care underwent lumbar interventional procedures. 2 patients underwent diagnostic procedures followed by surgery and are excluded in the analysis of interventional outcomes. This resulted in a remaining 33 patients. In these patients, the MCID for VAS was reached in 48% ((16/33), 95% CI [31, 66]), MCID for ODI was reached in 45% ((15/33), 95% CI [28, 62]), MCID for VAS and ODI was reached in 39% ((13/33), 95% CI [23, 56]) and the MCID for VAS or ODI was reached in 55% ((18/33), 95% CI [23, 56]), (Fig. 9).

Of the interventional low-back pain patients, one was lost to follow-up after their procedure and was considered a treatment failure. Of the remaining 34, 7 were discharged with treatment recommendations, 18 were discharged after successful procedures, 7 underwent surgery after failed therapeutic procedures and 2 underwent surgery after positive diagnostic procedures. Like the cervicothoracic interventional treatment failures, failed lumbar interventional patients did not undergo recommended follow-up procedures.

There was a total of 9 lumbar surgical patients in this series. All 9 surgical patients reached MCID for VAS and ODI together.

## 4 Discussion

### 4.1 Feasibility of telehealth

Telehealth feasibility employing a satellite telehealth capable facility has been established in studies involving orthopedic patients published prior to the pandemic emergency [[2], [3], [4],^6^]. Despite that, the use of in-home telehealth services was limited prior to the pandemic and not widely utilized [32]. With the pandemic, the need to minimize exposure to SARS-CoV-2 began to influence provider attitudes about telehealth and increase utilization [7,10]. Still, some providers limited their telehealth services to communication based follow-ups, like reviewing MRI results or checking on post-op patients [33]. One of the most prolific reported spine telehealth utilization rates come from the University of Pennsylvania, where during a 4-week period spanning from March to April 2020, the group executed 695 home-based telehealth evaluations, representing about 93% of all consultations [12]. Although our patients are fewer, our 97% success in executing home-based telehealth consultations during the first 6 months of the pandemic mirrors such a high utilization rate.

### 4.2 In-person follow-ups

The 26% rate of in-person follow-ups in our study mostly reflects our protocol of examining patients in-person prior to any planned procedures. This approach, while rarely changing procedural plans [34] is commonly accepted [10,16]. In our case series, the difference between our telehealth high initial consultation and moderate follow-up rate is largely attributable to this practice.

### 4.3 Accuracy in planning interventions

In our study, there was a very high degree of accuracy in telehealth-based procedure planning. Following an in-person evaluation on the day of a planned procedure, 100% (42/42) of completed interventional procedures in patients with pre-operative telehealth visits were consistent with the planned procedure. A similar accuracy rate in spine intervention procedure planning using telehealth has been reported in the literature [34]. However, In-person examination in our series was required to inform laterality and levels injected in the case of cervical, thoracic or lumbar facet blocks. While it has been reported that in-person evaluation after telehealth planning can commonly result in a change in the levels planned for surgery [18], we did not find that to be the case. Our accuracy rate in telehealth planning of spine surgical procedures was high (100% (9/9)). This accuracy rate in telehealth spine surgical planning is higher than the 80%-94% rates reported in the literature [17,18,35]. The likely explanation may lie in the fact that interventional procedures were performed on all patients prior to consideration of surgery. As a result, this cohort would become well known to the primary operator (GR). Another explanation might lie in the relatively small number of surgical patients in our series.

### 4.4 Cervicothoracic outcomes

Among patients presenting with cervicothoracic pain in the present study, 89% of injuries were because of a motor vehicle accident. As such, neck and upper back pain cases in our series were overwhelmingly due to whiplash associated disorder (WAD). Our overall treatment success rate, as noted by reaching MCID for NDI or VAS by discharge, was 71% (53/75). Although direct comparisons are difficult due to differing methodology and outcome measures, these results are generally comparable with the pre-pandemic and non-telehealth literature. In a review conducted by The Bone and Joint Decade 2000-2010 Task Force on Neck Pain and its Associated Disorders, it was noted that approximately 50% of those with WAD report neck pain 1 year after their injuries [36]. Similarly, a randomized trial comparing pragmatic treatment (medications, physical therapy and counseling) to usual care found that after 12 months follow-up, non-recovery rates in the pragmatic and usual care arms was 64% and 49%, respectively [37]. The rates of persistent neck symptoms in our series are higher than those reported by Lamb, et al., in the Managing Injuries of the Neck Trial (MINT) [38]. However, the cohorts from MINT STEP-1 suffered from a high lost to follow-up rate and, unlike our series, were reported to have relatively low average disability scores. The MINT STEP-2 cohorts harbored higher degrees of neck disability and pain, as in our series. The mean change in NDI in MINT STEP-2 was similar to that seen in our series.

### 4.5 Cervicothoracic conservative care

It is difficult to make inferences regarding the telehealth supervision of conservative care for cervicothoracic pain in this study. Our case series had a heterogenous time to presentation, with a significant number of patients presenting in the subacute or chronic period where later presentations are expected to do more poorly. Additionally, as a specialized spine referral center, our patients tended to have higher pain and disability levels than the general WAD population. Furthermore, during the early SARS-CoV-2 pandemic emergency our usual and customary prescribed conservative care consisting of in-person physical or chiropractic therapy was not available to many patients. Given those difficulties, our categorical conservative care success rate for reaching MCID VAS or NDI compares well with the conservative care literature [37,[39], [40], [41]].

It should be noted that the participation rates in formal therapy in our study were not recorded, and our late presenting patients may have undergone therapy in the pre-pandemic era, thus resulting in a heterogenous population. Varying forms of conservative care may lead to similar results, according to published literature. Several large reviews have shown that some treatments, such as manipulation and supervised therapy, are as effective, or no better than over the counter medications, exercise, education and self-care advice [[42], [43], [44]]. Given the mixed results of studies comparing home based care to formal therapy, it is not surprising that overall patient conservative care outcomes might not be affected by a particular modality of conservative care.

### 4.6 Interventional cervicothoracic treatment outcomes

WAD is commonly associated with cervical facet injury [[45], [46], [47]]. As expected, cervicothoracic interventions in our series were heavily weighted towards facet interventions, with 75% (18/24) of interventions being cervicothoracic medial branch blocks and the rest being epidural injections. Despite these interventions, we were successful in only 63% (15/24) of cases. The likely reason is that although the study operator (GR) follows published guidelines on selecting cervical or thoracic facet injury patients for rhizotomy based on medial branch blocks [48], 39% (7/18) of patients who underwent diagnostic cervicothoracic medial branch blocks deemed positive did not undergo the subsequently recommended therapeutic rhizotomy procedure. These patients would not be expected to improve. The reasons for non-compliance are poorly understood but may relate to pandemic-related difficulty in scheduling or performing procedures.

### 4.7 Lumbar outcomes

The natural history and course of patients with low-back pain is a difficult one, with many patients suffering from chronic or recurrent symptoms. Several studies have shown that persistent low-back pain continues to be reported at 1–7 years follow-up in 28%–75% of respondents [[49], [50], [51], [52]]. Persistent or recurrent symptoms are common even in patients who undergo interventional treatment [53]. Although direct comparisons with the reported literature are difficult due to differing methodology and outcome measures, our overall categorical success rate of 70% (56/80) compares favorably with the pre-pandemic and non-telehealth literature on patients presenting for medical care after an episode of low-back pain.

### 4.8 Lumbar conservative care

Like the case of cervicothoracic conservative care in this series, it is difficult to make inferences regarding the telehealth supervision of conservative care due to the heterogenous time to presentation, our being a specialized spine referral center with a higher degree of morbidity among patients and the lack of our previous usual and customary conservative care during the early SARS-CoV-2 pandemic. Given those difficulties, we achieved a 36% (29/80) conservative care success rate for treating low back pain. While comparisons are difficult due to methodology and modalities, our conservative care success rate approaches what has been previously reported [54,55].

Numerous studies have been published on the effectiveness of home-based care in low back pain. The Cherkin study population probably is most similar to our early pandemic patients in that they were treated primarily via education [55] yet results were very similar to physical therapy and manipulation. In studies of sub-acute and chronic low back pain, several studies showed little or no overall difference in outcomes after adding chiropractic care or manipulation therapy to usual or home care [56,57] and one study found no benefit in most after 52 weeks [58]. Similar findings were reflected in the 2020 North American Spine Society guidelines for the treatment of non-specific low back pain, where spinal manipulative therapy in acute low back pain should be expected to result in similar outcomes to no treatment, medication or modalities [59]. As in the case of our cervicothoracic patients, our low back pain patient conservative care results, despite their heterogeneity in the use of formal supervised therapy, mirror what one would expect based on the literature.

### 4.9 Lumbar interventional procedures

As reported above, the telehealth determination of the planned lumbar intervention survived the in-person pre-procedure examination in all cases. Of 39 lumbar interventional procedures performed on 35 patients, 74% (29/39) were epidural injections and 26% (10/39) were lumbar medial branch blocks. In our series, the success rate for lumbar epidural injections was 48% ((14/29), 95% CI [30, 66]). Excluding 2 patients who underwent surgery after a positive diagnostic test, interventional procedures were successful in treating pain or disability in 55% (18/33) of patients. Treatment failures included 1 patient lost to follow-up after intervention and 7 patients discharged after declining a recommendation for surgery following interventional procedures. As in the cervicothoracic patients in this series, the reasons for non-compliance are poorly understood but may relate to pandemic related difficulty in scheduling or performing procedures.

### 4.10 Lumbar surgeries

Telehealth was successfully utilized to determine surgical candidates and to determine surgical success. All 9 surgical cases in our series were successful. The 9% (9/102) surgical rate in our series is higher than that reported in a prospective cohort of 392 patients followed over 5 years by Wendelien, et al., who found that 5% (18/392) of patients ultimately would undergo spinal surgery [49], but also substantially less than the 49% (19/39) reported by Kennedy, et al., in his prospective cohort study of the long term outcomes of patients undergoing epidural injections [53]. It is also lower than the 32% surgical cross over rate seen in a recent study of 136 patients randomized to prolonged conservative care [60].

## 5 Strengths and limitations

The present study has several strengths. It is one of the few papers describing outcomes in spinal telehealth during the early SARS-CoV-2 pandemic emergency. Another strength is that the study contains categorical as well as continuous outcomes data. There are also limitations to the current study. This is a retrospective case series, with all its attendant risks. However, selection bias was limited by enrolling all consecutive patients during the first six-month period of the pandemic. Accuracy and availability of the medical record was not an issue due to the availability of electronic records. A control cohort, due to the pandemic emergency, would not have been possible, less it was a historical control. While a historical cohort would no doubt have improved the quality of this study, it was outside of the study design and felt to be overly burdensome because a cohort would have to have been reported for each treatment modality and each spinal pain region. Another limitation is that the pandemic emergency resulted in a lack of availability in health care resources, such that our look at telehealth outcomes might be more reflective of the pandemic than a normal period. For example, although we do not have specific data on the proportion of patients who did not attend formal therapy or had their MRI’s delayed, it is well known that such services were scarcely available during the first 2 months of the study period. Additionally, needed procedures were delayed during the same period, potentially prolonging patient pain and disability. Another concern is that patient compliance with recommended therapeutic procedures may have been affected over concerns of viral exposure, also effecting outcomes. An additional limitation is our heterogenous patient population. This was a subacute as well as a chronic pain population and the outcomes were likely skewed by the chronic patients. Lastly, the study has a relatively short follow-up period of a mean of 79 days.

## 6 Conclusion

Telehealth in our series was highly feasible and easily deployable. Telehealth allowed an accurate way to monitor patient care, determine conservative care outcomes and determine candidacy for follow-up interventions or surgery. Telehealth examination protocols were sufficient to screen patients with serious deficits requiring a higher level of care. When procedures were performed, telehealth resulted in a feasible and accurate way to plan and monitor the outcomes of those procedures. Patient follow-up was maintained by a hybrid approach, heavily reliant on telehealth. Overall, the outcomes for patients with cervicothoracic and low-back pain in this series are comparable to that reported in the pre-pandemic and non-telehealth literature. The authors are confident that the future of telehealth in spinal health has been secured and that telehealth resources will continue to be widely utilized in the post-pandemic period.

## Data availability statement

The data that supports the findings of this study is available at http://doi.org/10.17632/2hk4kmv377.1.

## Funding statement

This research received no specific grant from any funding agency in the public, commercial, or not-for-profit sectors.

## Declaration of competing interest

None.
